# Supplementary material for: Neurogenesis mediated plasticity is associated with reduced neuronal activity in CA1 during context fear memory retrieval
Source: Sci Rep. 2022 Apr 29;12:7016. doi: 10.1038/s41598-022-10947-w (PMC9054819; doi:10.1038/s41598-022-10947-w)
Supplement: Supplementary file 1 — Supplementary Legends. [file 41598_2022_10947_MOESM1_ESM.docx]

**Supplementary Figures**


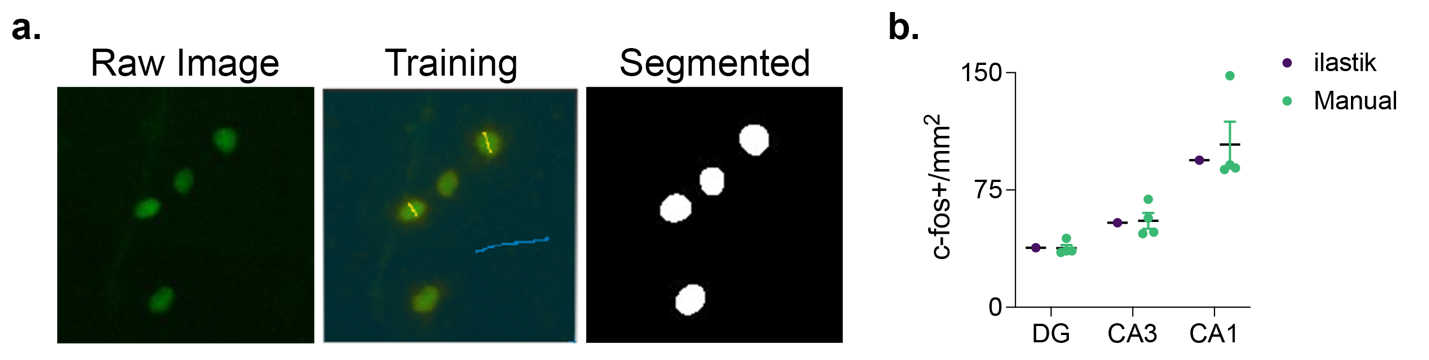


**Supplementary Figure S1 | Semi-automated c-fos+ cell segmentation using the supervised machine learning-based tool *ilastik.*** (**a**) Using supervised machine learning, the user interactively defined the criteria by which *ilastik* segmented c-fos+ cells from photomicrographs. (**b**) The segmentation of c-fos+ cells by *ilastik* yielded counts which were within the 95% confidence intervals of densities reported through independent hand scoring (*n* = 4) of the same image sets in the dentate gyrus, CA3, and CA1. One outlier was identified in the manual counts for area CA1 using a Grubb’s analysis. We chose to leave this data point in as it is part of the distribution of user-generated counts. Removal of this outlier would bring the manual counts into closer agreement with the *ilastik*-generated count. Data shown are mean ± 95% CI.

**
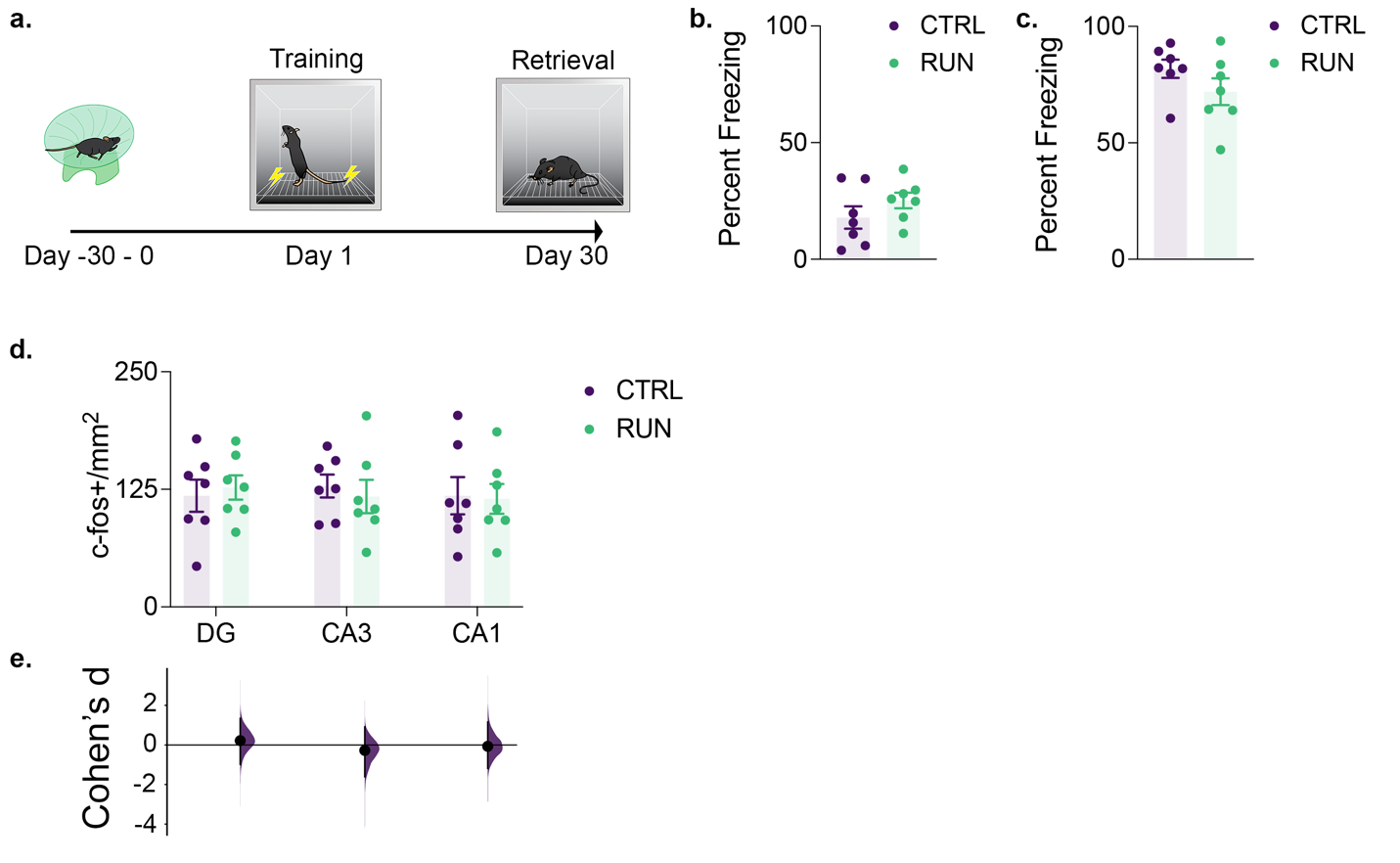
**

**Supplementary Figure S2 | The retrieval and underlying hippocampal activity of memories acquired after running-induced increased neurogenesis is not altered.** (**a**) After 30 days of running wheel access (*n* = 7) or conventional housing (*n* = 7), mice were contextually fear conditioned. Contextual memory retrieval was assessed 30 days later during context reintroduction. During both (**b**) training and (**c**) retrieval, prior running wheel access did not alter the proportion of the trial spent freezing. (**d**) The density of c-fos+ cells tagged to context reintroduction in the dentate gyrus, CA3, and CA1 did not change with prior running wheel access. (**e**) The magnitudes of effect sizes (Cohen’s d) of the differences in c-fos+ cells in all three of the examined hippocampal subregions did not differ from a bootstrapped 95% confidence interval. Data analysis used Two-Sample T-Test (**b**,**c**), ANOVA (**d**) with Tukey’s test during *post-hoc* multiple comparisons, and Multiple Two-Groups estimation statistics with Cohen’s d as a measure of effect size (**h**). **P* < 0.05. Data shown are mean ± s.e.m. See Supplementary Table S5 for full statistical analysis

**
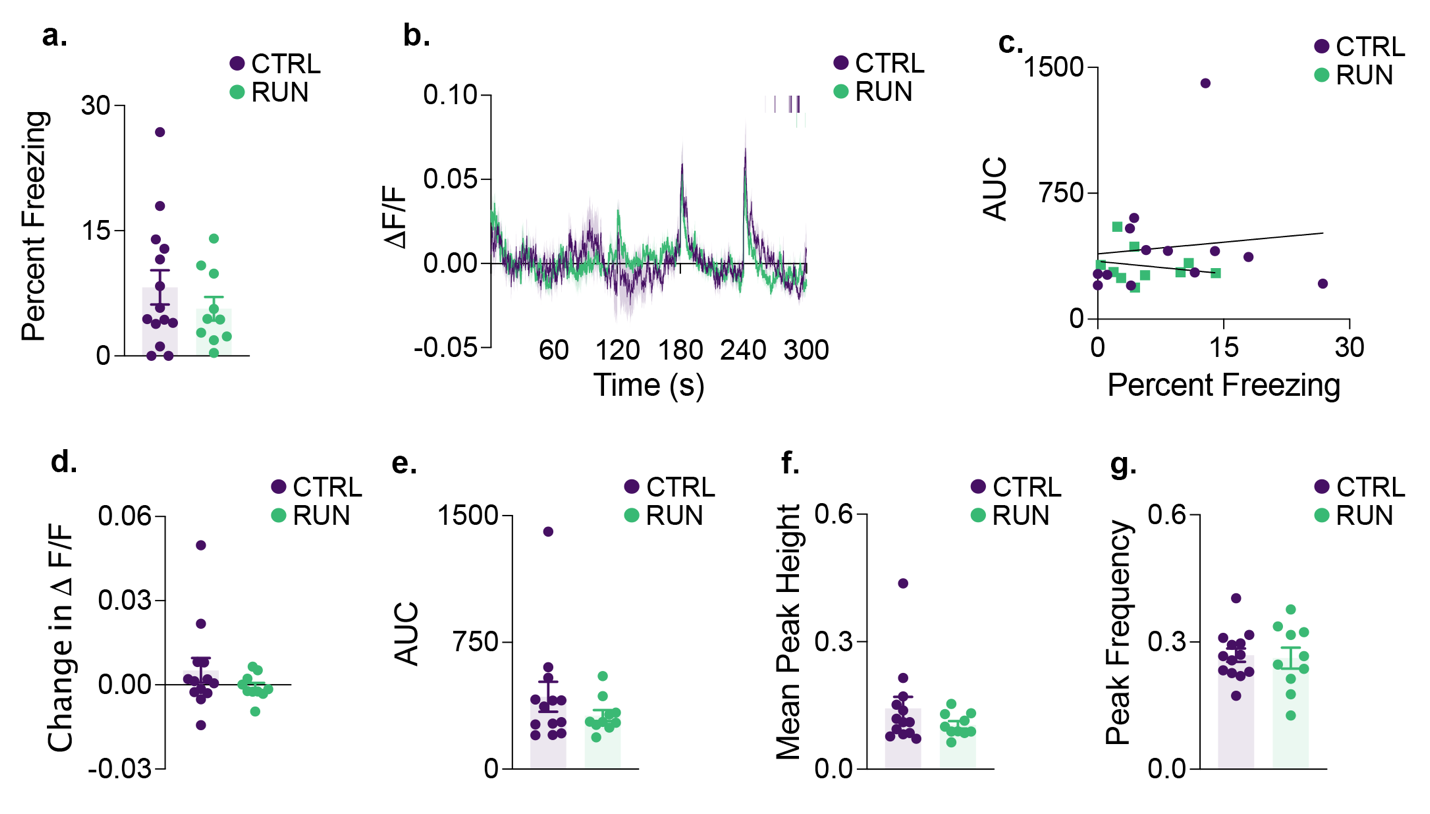
**

**Supplementary Figure S3 | CA1 activity does not differ during contextual conditioning prior to running-induced changes in neurogenesis.** (**a**) During contextual fear conditioning prior to running wheel access (*n* = 10) or conventional housing (*n* = 14), there was no difference in the extent to which mice froze during the trial. (**b**) Mean GCaMP7f fluorescence across the context reintroduction trial with the mode of group freezing plotted above. (**c**) The area under the curve of the GCaMP7f fluorescence curve did not correlate with percent freezing in either group during this training trial. (**d**) When mice were transferred from the home cage to the conditioning chambers, neither group showed a significant change in mean GCaMP7f fluorescent signal. Throughout the training trial, neither group differed in (**e**) overall area under the GCaMP7f fluorescent curve, (**f**) mean photometry signal peak height, or (**g**) the frequency of peaks in the photometry signal. Data analysis used Two-Sample T-Test (**a**,**d,e,f,g**). **P* < 0.05. Data shown are mean ± s.e.m. See Supplemental Table S6 for full statistical analysis


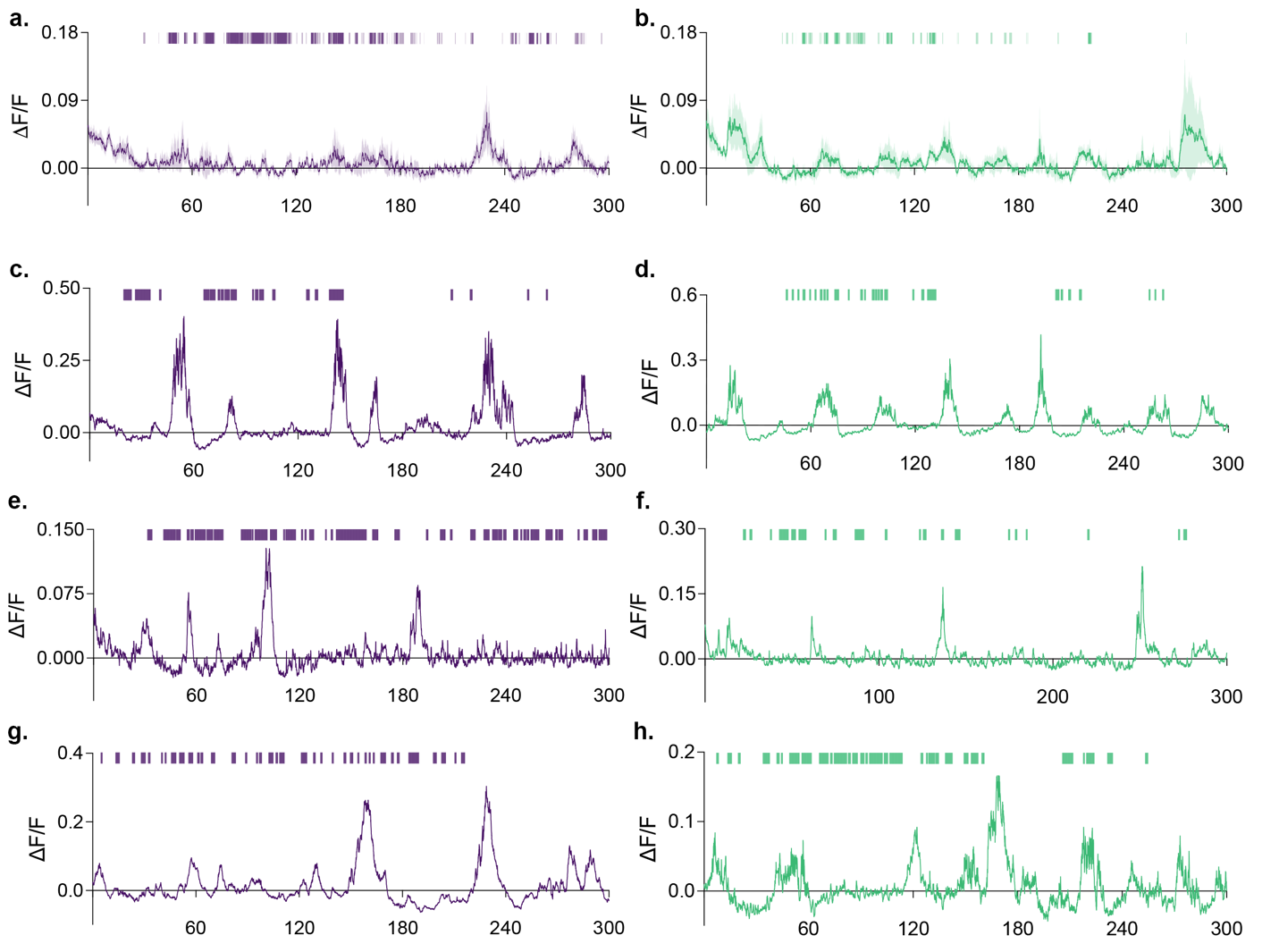


**Supplementary Figure S4 | Non-baseline corrected group and representative individual recordings of CA1 activity during contextual memory retrieval.** Group mean traces from control (**a**, *n* = 8) and runners (**b**, *n* = 7) prior to baseline correction, with median group freezing plotted above. Representative individual traces and instantaneous freezing from control (**c**,**e**,**g**) and running (**d**,**f**,**h**) groups. Data shown are mean ± s.e.m.
