# Supplementary material for: Neurogenesis mediated plasticity is associated with reduced neuronal activity in CA1 during context fear memory retrieval
Source: Sci Rep. 2022 Apr 29;12:7016. doi: 10.1038/s41598-022-10947-w (PMC9054819; doi:10.1038/s41598-022-10947-w)
Supplement: Supplementary file 6 — Supplementary Table S1. [file 41598_2022_10947_MOESM6_ESM.docx]

**Supplementary Table S1: Statistics for the comparisons outlined in Figure 1.**

| **Two-Sample T Test, two-tailed** | | |  |  |  |  |
| --- | --- | --- | --- | --- | --- | --- |
| Figure | x-axis | y-axis | Groups (*n*) | p-value | t stat; df | Cohen’s *d* |
| **b** | Treatment Group | Percent Freezing | CTRL (14); RUN (14) | 0.1434 | t=1.509, df=26 | -0.57 |
| **c** | Treatment Group | Percent Freezing | CTRL (14); RUN (14) | 0.0005 | t=3.995, df=26 | -1.51 |
| **e** | Treatment Group | DCX+/mm^2^ | CTRL (14); RUN (14) | 0.0014 | t=3.574, df=26 | 1.35 |
| **Two-Factor ANOVA** | | | |  |  |  |
| Panel | x-axis | y-axis | Factor/*Comparison* | p-value | F stat; df | Cohen’s *d* |
| **g** | Region | c-fos+/mm^2^ | Interaction (*Tukey*) | 0.0114 | F (2, 72) = 4.768 |  |
|  |  |  | *CA1:Sedentary vs. CA1:Running* | 0.0214 |  | 0.34 |
|  |  |  | *CA3 :Sedentary vs. CA3 :Running* | 0.6243 |  | -1.17 |
|  |  |  | *DG:Sedentary vs. DG:Running* | 0.884 |  | -1.53 |
|  |  |  | Areas of Hippocampus | <0.0001 | F (2, 72) = 12.09 |  |
|  |  |  | Treatment | 0.0353 | F (1, 72) = 4.602 |  |
