# Supplementary material for: Neurogenesis mediated plasticity is associated with reduced neuronal activity in CA1 during context fear memory retrieval
Source: Sci Rep. 2022 Apr 29;12:7016. doi: 10.1038/s41598-022-10947-w (PMC9054819; doi:10.1038/s41598-022-10947-w)
Supplement: Supplementary file 7 — Supplementary Table S2. [file 41598_2022_10947_MOESM7_ESM.docx]

**Supplementary Table S2: Statistics for the comparisons outlined in Figure 2.**

| **Two-Sample T Test, two-tailed** | | |  |  |  |  |
| --- | --- | --- | --- | --- | --- | --- |
| Panel | x-axis | y-axis | Groups (*n*) | p-value | t stat; df | Cohen’s *d* |
| **c** | Treatment Group | DCX+/mm^2^ | CTRL (14); RUN (10) | 0.0417 | t=2.162, df=22 | 0.895 |
| **e** | Treatment Group | Percent Freezing | CTRL (14); RUN (10) | 0.0454 | t=2.121, df=22 | -0.878 |
| **g** | Treatment Group | ΔF/F | CTRL (14); RUN (10) | 0.9625 | t=0.04761, df=22 | 0.0197 |
| **h** | Treatment Group | Change in ΔF/F | CTRL (14); RUN (10) | 0.0237 | t=2.429, df=22 | -1.01 |
| **Pearson Correlation** | | |  |  |  |  |
| Panel | x-axis | y-axis | Groups (*n*) | p-value | correlation coefficient |  |
| **f** | Percent Freezing | DCX+/mm^2^ | CTRL (14) | 0.6457 | Pearson r=0.1349 |  |
|  |  |  | RUN (10) | 0.0106 | Pearson r = -0.7610 |  |
| **j** | Percent Freezing | AUC | CTRL (14) | <0.0001 | Pearson r = 0.9980 |  |
|  |  |  | RUN (10) | <0.0001 | Pearson r = 0.9933 |  |
| **Two-Factor ANOVA** | | | |  |  |  |
| Panel | x-axis | y-axis | Factor/*Comparison* | p-value | F stat; df | Cohen’s *d* |
| **k** | Behaviour | AUC | Interaction | 0.8959 | F (1, 44) = 0.01733 |  |
|  |  |  | Behaviour | 0.9164 | F (1, 44) = 0.01116 |  |
|  |  |  | Treatment Group | 0.0046 | F (1, 44) = 8.908 |  |
| **l** | Behaviour | Mean Peak Height | Interaction | 0.2856 | F (1, 44) = 1.168 |  |
|  |  |  | Behaviour | 0.3867 | F (1, 44) = 0.7645 |  |
|  |  |  | Treatment Group | 0.1135 | F (1, 44) = 2.607 |  |
| **m** | Behaviour | Peak Frequency | Interaction (*Tukey*) | 0.0039 | F (1, 44) = 9.297 |  |
|  |  |  | *Freezing:CTRL vs. Moving:CTRL* | <0.0001 |  | 1.67 |
|  |  |  | *Freezing:RUN vs. Moving:RUN* | 0.0066 |  | 0.216 |
|  |  |  | *Moving:CTRL vs. Moving:RUN* | 0.0177 |  | -0.979 |
|  |  |  | *Freezing:CTRL vs. Freezing:RUN* | 0.6139 |  | 0.931 |
|  |  |  | Row Factor | 0.0009 | F (1, 44) = 12.72 |  |
|  |  |  | Column Factor | 0.1952 | F (1, 44) = 1.730 |  |
