# Supplementary material for: Neurogenesis mediated plasticity is associated with reduced neuronal activity in CA1 during context fear memory retrieval
Source: Sci Rep. 2022 Apr 29;12:7016. doi: 10.1038/s41598-022-10947-w (PMC9054819; doi:10.1038/s41598-022-10947-w)
Supplement: Supplementary file 8 — Supplementary Table S3. [file 41598_2022_10947_MOESM8_ESM.docx]

**Supplementary Table S3: Statistics for the comparisons outlined in Figure 3.**

| **Two-Factor ANOVA** | | | |  |  |  |
| --- | --- | --- | --- | --- | --- | --- |
| Panel | x-axis | y-axis | Factor/*Comparison* | p-value | F stat; df | Cohen’s *d* |
| **c** | Region | PNN+/mm^2^ | Interaction (*Tukey*) | 0.0842 | F (2, 39) = 2.638 |  |
|  |  |  | *DG:CTRL vs. DG:RUN* | 0.9999 |  | -0.408 |
|  |  |  | *CA3:CTRL vs. CA3:RUN* | 0.9947 |  | -0.409 |
|  |  |  | *CA1:CTRL vs. CA1:RUN* | 0.031 |  | -1.05 |
|  |  |  | Region | <0.0001 | F (2, 39) = 59.95 |  |
|  |  |  | Treatment Group | 0.0275 | F (1, 39) = 5.248 |  |
| **Two-Sample T Test, two-tailed** | | |  |  |  |  |
| Panel | x-axis | y-axis | Groups (*n*) | p-value | t stat; df | Cohen’s *d* |
| **f** | Treatment Group | Contiguity | CTRL (8); RUN (7) | 0.0038 | t=3.511, df=13 | -1.82 |
| **Pearson Correlation** | |  |  |  |  |  |
| Panel | x-axis | y-axis | Groups (*n*) | p-value | correlation coefficient |  |
| **g** | DCX+/mm^2^ | CA1 PNN+/mm^2^ | CTRL (8) | 0.0844 | Pearson r=-0.6446 |  |
|  |  |  | RUN (7) | 0.5586 | Pearson r=-0.2697 |  |
| **h** | DCX+/mm^2^ | Contiguity | CTRL (8) | 0.0423 | Pearson r=-0.7241 |  |
|  |  |  | RUN (7) | 0.4492 | Pearson r=-0.3445 |  |
