# Supplementary material for: Neurogenesis mediated plasticity is associated with reduced neuronal activity in CA1 during context fear memory retrieval
Source: Sci Rep. 2022 Apr 29;12:7016. doi: 10.1038/s41598-022-10947-w (PMC9054819; doi:10.1038/s41598-022-10947-w)
Supplement: Supplementary file 9 — Supplementary Table S4. [file 41598_2022_10947_MOESM9_ESM.docx]

**Supplementary Table S4: Statistics for the comparisons outlined in Figure 4.**

| **Two-Factor ANOVA** | | | |  |  |  |
| --- | --- | --- | --- | --- | --- | --- |
| Panel | x-axis | y-axis | Factor/*Comparison* | p-value | F stat; df | Cohen’s *d* |
| **a** | Region | PNN+/mm^2^ | Interaction (*Holm-Šídák*) | <0.0001 | F (4, 36) = 10.86 |  |
|  |  |  | *CA1:TMZ vs. CA1:CTRL* | 0.0004 |  | -2.43 |
|  |  |  | *CA1:CTRL vs. CA1:MEM* | 0.0419 |  | -3.11 |
|  |  |  | *DG:TMZ vs. DG:CTRL* | 0.874 |  | -1.14 |
|  |  |  | *DG:CTRL vs. DG:MEM* | >0.9999 |  | -0.237 |
|  |  |  | *CA3:TMZ vs. CA3:CTRL* | 0.8216 |  | 0.875 |
|  |  |  | *CA3:CTRL vs. CA3:MEM* | 0.6997 |  | -1.03 |
|  |  |  | Region | <0.0001 | F (2, 36) = 21.87 |  |
|  |  |  | Treatment Group | <0.0001 | F (2, 36) = 18.16 |  |
| **One-Factor ANOVA** | | | |  |  |  |
| Panel | x-axis | y-axis | Factor/*Comparison* | p-value | F stat; df | Cohen’s *d* |
| **b** | Treatment Group | DCX+/mm^2^ | ANOVA (*Holm-Šídák)* | 0.0009 | F (2, 12) = 13.39 |  |
|  |  |  | *CTRL vs. TMZ* | 0.0366 |  | -3.25 |
|  |  |  | *CTRL vs MEM* | 0.0366 |  | 1.36 |
| **c** | Treatment Group | PNN+/mm^2^ | ANOVA (*Tukey*) | 0.0005 | F (2, 11) = 16.78 |  |
|  |  |  | *4 vs. 8* | 0.0161 |  | 1.82 |
|  |  |  | *4 vs. 12* | 0.0004 |  | 3.29 |
|  |  |  | *8 vs. 12* | 0.0198 |  | 3.17 |
| **d** | Treatment Group | DCX+/mm^2^ | ANOVA (*Tukey*) | <0.0001 | F (2, 11) = 52.60 |  |
|  |  |  | *4 vs. 8* | 0.0126 |  | -2.1 |
|  |  |  | *4 vs. 12* | <0.0001 |  | -9.81 |
|  |  |  | *8 vs. 12* | <0.0001 |  | -3.9 |
| **Two-Sample T Test, two-tailed** | | |  |  |  |  |
| Panel | x-axis | y-axis | Groups (*n*) | p-value | t stat; df | Cohen’s *d* |
| **f** | Treatment Group | Percent Freezing | CTRL (4); RUN (4) | 0.0046 | t=4.397, df=6 | -2.25 |
| **g** | Treatment Group | PNN+/mm^2^ | CTRL (4); RUN (4) | <0.0001 | t=9.937, df=6 | -7.03 |
| **h** | Treatment Group | DCX+/mm^2^ | CTRL (4); RUN (4) | 0.9259 | t=0.09694, df=6 | -0.0685 |
