# Supplementary material for: Neurogenesis mediated plasticity is associated with reduced neuronal activity in CA1 during context fear memory retrieval
Source: Sci Rep. 2022 Apr 29;12:7016. doi: 10.1038/s41598-022-10947-w (PMC9054819; doi:10.1038/s41598-022-10947-w)
Supplement: Supplementary file 10 — Supplementary Table S5. [file 41598_2022_10947_MOESM10_ESM.docx]

**Supplementary Table S5: Statistics for the comparisons outlined in Supplemental Figure S2.**

| **Two-Sample T Test, two-tailed** | | |  |  |  |  |
| --- | --- | --- | --- | --- | --- | --- |
| Panel | x-axis | y-axis | Groups (*n*) | p-value | t stat; df | Cohen’s *d* |
| **b** | Treatment Group | Percent Freezing | CTRL (7); RUN (7) | 0.2344 | t=1.252, df=12 | 0.669 |
| **c** | Treatment Group | Percent Freezing | CTRL (7); RUN (7) | 0.185 | t=1.406, df=12 | -0.752 |
| **Two-Factor ANOVA** | | | |  |  |  |
| Panel | x-axis | y-axis | Factor/Comparison | p-value | F stat; df | Cohen’s *d* |
| **d** | Region | c-fos+/mm^2^ | Interaction | 0.8394 | F (3, 48) = 0.2802 |  |
|  |  |  | Region | 0.908 | F (3, 48) = 0.1822 |  |
|  |  |  | Treatment Group | 0.7853 | F (1, 48) = 0.07507 |  |
