# Supplementary material for: Neurogenesis mediated plasticity is associated with reduced neuronal activity in CA1 during context fear memory retrieval
Source: Sci Rep. 2022 Apr 29;12:7016. doi: 10.1038/s41598-022-10947-w (PMC9054819; doi:10.1038/s41598-022-10947-w)
Supplement: Supplementary file 11 — Supplementary Table S6. [file 41598_2022_10947_MOESM11_ESM.docx]

**Supplementary Table S6: Statistics for the comparisons outlined in Supplemental Figure S3.**

| **Two-Sample T Test, two-tailed** | | |  |  |  |  |
| --- | --- | --- | --- | --- | --- | --- |
| Panel | x-axis | y-axis | Groups (*n*) | p-value | t stat; df | Cohen’s *d* |
| **a** | Treatment Group | Percent Freezing | CTRL (14); RUN (10) | 0.3574 | t=0.9401, df=22 | -0.389 |
| **d** | Treatment Group | Change in ΔF/F | CTRL (13); RUN (10) | 0.2685 | t=1.137, df=21 | -0.478 |
| **e** | Treatment Group | AUC | CTRL (13); RUN (10) | 0.3031 | t=1.056, df=21 | -0.444 |
| **f** | Treatment Group | Mean Peak Height | CTRL (13); RUN (10) | 0.2358 | t=1.221, df=21 | -0.513 |
| **g** | Treatment Group | Peak Frequency | CTRL (13); RUN (10) | 0.8067 | t=0.2478, df=21 | -0.104 |
| **Pearson Correlation** | |  |  |  |  |  |
| Panel | x-axis | y-axis | Groups (*n*) | p-value | correlation coefficient |  |
| **c** | Percent Freezing | AUC | CTRL (13) | 0.7107 | Pearson r=-0.1140 |  |
|  | Percent Freezing | AUC | RUN (10) | 0.5615 | Pearson r=-0.2094 |  |
